# Undergraduate medical education in India and scope of pediatric surgery teaching

**DOI:** 10.4103/0971-9261.57697

**Published:** 2009

**Authors:** Biswanath Mukhopadhyay

**Affiliations:** Department of Pediatric Surgery, N R S Medical College and Hospital, Kolkata, India E-mail: drbmukhopadhyay@yahoo.co.in

The Medical Council of India (MCI) was established as a statutory body under the provisions of the Indian Medical Council Act, 1933, which was later repealed by the Indian Medical Council Act, 1956, with minor amendments in 1958 and 1964. Since then, several amendments have been made with the main interest to maintain uniform standards of medical education at undergraduate and postgraduate level.[1] Salient features of regulations on undergraduate medical education in India are published in part III, section 4 of the Gazette of India, 17^th^ May, 1997 and the last amendment was made on 22.12.08 as seen on MCI website on 31.08.2009.[1]

Undergraduate medical education in India takes five-and-a-half years, including one year of compulsory internship. The period of four-and-a-half years (nine semesters, six months each) is divided into three phases. Each semester consists of approximately 120 teaching days of eight hours each college working time, including one hour of lunch.

## Phase I (two semesters):

Pre-clinical subjects: Anatomy, Physiology (including Biophysics), Biochemistry and introduction to community medicine (including humanities)

## Phase II (three semesters):

Para-clinical (Pathology, Pharmacology, Microbiology, Forensic Medicine including Toxicology and Community Medicine) and clinical subjects concurrently.

## Phase III (continuation of clinical subjects for seven semesters after passing phase I):

Medicine and allied specialties, Surgery and its allied specialties, Obstetric and Gynecology and Community Medicine; Pediatric including Neonatology.

Following are the guidelines related to Pediatric Surgery in the MCI curriculum

**Under the heading** Objectives in[1] the curriculum of Surgery (including Pediatric Surgery) are:

1. Etiology, pathophysiology, principles of diagnosis and management of common surgical problems including emergencies, in adults and children.

**Under the heading Skills,**[1] at the end of the course, the student should be able to:

1.Diagnose common surgical conditions, both acute and chronic, in adults and children.

5.Monitor patients of head, chest, spinal and abdominal injuries, both in adults and children.

9.  Diagnose neonatal and pediatric surgical emergencies and provide sound primary care before referring the patients to secondary / tertiary centers.

10.  Identify congenital anomalies and refer them for appropriate management.

In the list of operations – desired to be observed / assisted / performed - there are operations performed mainly by pediatric surgeons.

In 1954, noted pediatrician Dr. K C Chaudhary wrote in his editorial that it is astounding of the Indian Medical Council and the Indian Universities are oblivious of the developments that have taken place even in the British Isles since this report was written in 1944. All British Universities except Oxford and Cambridge now have a Department on Child Health. He also mentioned that in a recent circular, the Medical Council of India included Pediatrics as an ancillary branch of medicine.[2]

As per the conditions laid down by MCI, a high power committee supervises the training program in each Medical College. Unfortunately, Pediatric Surgery never got its due recognition in undergraduate medical education in India. The matter was discussed several times in the past in IAPS executive committees. A committee was formed (Dr. D K Gupta, AIIMS; Dr. K L N Rao, PGI; Dr. B Mukhopadhyay, Kolkata; Dr. Anirudh Shah, Ahmedabad; Dr. Bipin Puri, Army and Dr. Sujit Choudhury, New Delhi) to pursue the matter with MCI. Dr. D K Gupta took a leading role to initiate and make a curriculum of Pediatric Surgery to teach at the MBBS level.

The committee recommended the following topics to Medical Council of India:

1. Introduction to Pediatric Surgery – pre and post operative care
2. Inguino-scrotal swellings
3. Principles of plastic surgery –cleft lip and palate, lymphangioma, hemangioma
4. Intestinal obstruction – intestinal atresia, meconium ileus
5. Megacolon – Hirschsprung's disease, acquired megacolon
6. Anorectal malformations
7. Management of respiratory distress of neonates (esophageal atresia and tracheoesophageal fistula, congenital diaphragmatic hernia, congenital cystic adenomatoid malformations and congenital lobar emphysema)
8. Malignancies of childhood (Wilms' tumor, Neuroblastoma, teratomas, rhabdomyosarcoma, lymphomas)
9. Congenital malformations of urinary tract (congenital cystic disease of the kidney, pelviureteric junction obstruction, vesicoureteric reflux, primary megaureters including prune belly syndrome, posterior urethral valve, exstrophy epispadias complex, hypospadias and intersex disorders)
10. Congenital malformations of nervous system (spina bifida, neurogenic bladder and hydrocephalus)
11. Jaundice of infancy (congenital biliary atresia, neonatal hepatitis and choledochal cyst)
12. Burns in children
13. Pediatric trauma (principles of management of pediatric trauma and trauma of abdomen, thoracic, genitourinary and nervous system)

Considering the high incidence of congenital malformations, increasing incidence of solid malignancies and trauma, the following recommendations were made to MCI:

1. Topics selected are only guidelines for undergraduate teaching (can be covered by lectures, ward teaching and OPD classes).
2. These should be taught by qualified pediatric surgeons.
3. Each Medical college should have a pediatric surgical unit not only for teaching but also for service.
4. Undergraduate students should be circulated to pediatric surgery for about two weeks to orient themselves with the common pediatric surgical problems.
5. Interns should be rotated for one week in pediatric surgery.
6. In case there is no pediatric surgery teaching faculty in a particular medical college with a responsibility to teach the undergraduates, following options may be taken: The Indian Association of Pediatric Surgeons would help the department with lectures on the above topics. Alternatively, the respective medical colleges should arrange the services of the pediatric surgeons available locally or from other Institutes/ medical colleges.

During the previous Asian Congress of Pediatric Surgery held in New Delhi, Dr. D K Gupta arranged a meeting with Dr. Manmohan Singh, Honorable Prime Minister of India. He led a delegation of pediatric surgeons from different parts of the world. Dr. D K Gupta and the present author represented India. Dr. Gupta submitted our recommendations to Honorable Prime Minister of India. Subsequently, on the invitation from MCI (Vide MCI-7(9)/ 2007-Med. /20022 of 13.11.2007), IAPS representatives (Dr. D K Gupta, Dr. K L N Rao and Dr. Bipin Puri) had a meeting with the Secretary of MCI and made an appeal to accept our recommendations. Recently (January, 2009) Dr. Subir Kumar Chatterjee met the Secretary of Medical Council of India and discussed the whole issue for kind consideration by MCI. Unfortunately, the matter is still pending. MCI regulates the allocation of time among disciplines, percentage of lecture time, required percentage of class attendance, types of electives, time distribution of internship components and even duration of the lunch break.[3] With continuous efforts Pediatric medicine, Orthopedics, Psychiatry have been incorporated in to the curriculum including rotational posting during internship.[4,5] We must continue to pursue the matter with MCI.

Pediatric Surgery should be included as a separate subject in the medical curriculum with independent evaluation similar to Pediatric Medicine, Orthopedics, ENT, etc. Merely expanding the curriculum is not sufficient. The examination process must be more relevant, practical oriented and these changes will eventually translate in to better patient care. According to the Kyoto declaration (2002), every child has the right to best medical and surgical care and needs to be treated only by qualified and trained pediatric surgeons.
